# Targeting Host Factors to Treat West Nile and Dengue Viral Infections

**DOI:** 10.3390/v6020683

**Published:** 2014-02-10

**Authors:** Manoj N. Krishnan, Mariano A. Garcia-Blanco

**Affiliations:** 1 Program in Emerging Infectious Diseases, Duke-NUS Graduate Medical School, Singapore 169857, Singapore; 2 Center for RNA Biology, Duke University Medical Center, Durham, NC 27710, USA; 3 Department of Molecular Genetics and Microbiology, Duke University Medical Center, Durham, NC 27710, USA; 4 Department of Medicine, Duke University Medical Center, Durham, NC 27710, USA

**Keywords:** West Nile virus, dengue virus, host factor, drug targets

## Abstract

West Nile (WNV) and Dengue (DENV) viruses are major arboviral human pathogens belonging to the genus *Flavivirus*. At the current time, there are no approved prophylactics (e.g., vaccines) or specific therapeutics available to prevent or treat human infections by these pathogens. Due to their minimal genome, these viruses require many host molecules for their replication and this offers a therapeutic avenue wherein host factors can be exploited as treatment targets. Since several host factors appear to be shared by many flaviviruses the strategy may result in pan-flaviviral inhibitors and may also attenuate the rapid emergence of drug resistant mutant viruses. The scope of this strategy is greatly enhanced by the recent *en masse* identification of host factors impacting on WNV and DENV infection. Excellent proof-of-principle experimental demonstrations for host-targeted control of infection and infection-induced pathogenesis have been reported for both WNV and DENV. These include exploiting not only those host factors supporting infection, but also targeting host processes contributing to pathogenesis and innate immune responses. While these early studies validated the host-targeting approach, extensive future investigations spanning a range of aspects are needed for a successful deployment in humans.

## 1. Introduction

West Nile virus (WNV) and the four dengue viruses (DENV1-4) are flaviviruses belonging to the *Flaviviridae* family [1]. Many mosquito-borne flaviviruses cause a wide range of severe diseases. The mosquito-borne cluster of flaviviruses cause either systemic manifestations (e.g., fever and hemorrhage) or primarily neurological damage (e.g., encephalitis). Yellow fever virus (YFV) and DENVs cause systemic illnesses whose outcomes range from mild to death, and manifestations include high fever, severe headache, retro-orbital pain, and rash [2]. Dengue fever (DF) can progress to dengue hemorrhagic fever (DHF) and dengue shock syndrome (DSS) [3]. The global health impact of the four DENV viruses is staggering: DENVs threaten half of the world’s population, and result in ~400 million infections and 15,000–30,000 deaths per year [4]. Among the neurotropic viruses, WNV and Japanese encephalitis infections can have systemic and also neurological manifestations; as many as 10% of WNV infected patients with neurological symptoms succumb to the disease [2]. 

All flaviviruses have a ~11 kb positive strand RNA genome and mRNA, which is translated into a single polyprotein that is cleaved into three structural and seven nonstructural (NS) proteins necessary for viral propagation [1]. The flaviviral lifecycle involves complex interactions with many of the host cell cytoplasmic and, very likely, nuclear structures and components. WNV and DENV1-4 enters cells via interactions with one or more receptors and co-receptors at the plasma membrane, delivers its genome to the cytoplasm, and sets up translation-replication-assembly factories in membranous structures associated with the endoplasmic reticulum. The assembled progeny virions will eventually leave the cells through secretory pathways, and initiate subsequent infections 

Given their genome’s limited coding capacity and their lifecycle’s complexity, it is not surprising that flaviviruses require scores of host factors [5,6]. These host factors are gene products (RNA or protein) that critically impact viral replication either positively (dependency or proviral factor) or negatively (restriction or antiviral factor). The identification of these host factors and the characterization of their interactions with viral proteins and RNAs are critical for the understanding of flaviviral replication, and should significantly inform our understanding of disease progression and pathogenesis. Moreover, each of these host factors is a candidate for therapeutic intervention. 

In this chapter we will provide a review on the current understanding of the host factors that impact replication of WNV and DENV focusing primarily on those factors we deem particularly druggable. As implied by this focus, our review will be emphasizing only those factors in the human (or mammalian) host. 

## 2. Approaches to Identify WNV and DENV Host Factors

*En masse* approaches have accelerated the discovery of host factors that impact propagation of WNV and DENV by interrogating tens of thousands of gene products or interactions simultaneously. While these methods have important limitations and should be considered hypotheses generating exercises that result in lists of candidate host factors that must be tested carefully, they have generated very important data. 

### 2.1. Methods that Identify Changes in Expression of Host RNAs and Proteins

Genome-scale analyses of transcript levels, using one of many approaches (e.g., next generation RNA sequencing), have revealed the identity of host mRNAs that significantly vary after WNV [7,8,9] and DENV infection [8,10,11,12,13,14,15,16,17]. Among the earliest of these studies Fink *et al.* (2007) identified three pathways containing many component genes with altered expression upon DENV infection of cells in culture and DENV infected blood samples: NF-kappaB initiated immune responses, type I interferon (IFN) and the ubiquitin proteasome pathway [13]. Among studies that examined changes in the transcriptome, Sessions *et al.* (2013) focused on the expression of mRNA isoforms (products of transcriptional and post-transcriptional events) and concluded that isoforms of genes implicated in the innate immune responses were differentially processed during infection with wild type and attenuated strains of DENV [16]. A few studies have looked at alterations in protein expression in WNV [6] or DENV infection [18,19,20,21]. Proteins associated with the IFN response and the proteasome were upregulated [19], consistent with transcriptomic studies above. A recent study addressed changes in activity of kinases by mass-spectrometry-based chemoproteomic profiling with reactive ATP- and ADP-acyl phosphates as probes, and identified DNA-dependent protein kinase (DNA-PK) as activated early in DENV infection [22].

The logic behind interrogating gene expression differences is the assumption that dependency or restriction factors will be overrepresented among factors with altered expression (or altered modification) and indeed several studies suggest that this is likely true [13,19]. 

### 2.2. Methods that Map the Viral Interactome

Many methods look globally for host gene products that physically interact with viral proteins or RNAs. Two-hybrid interaction screens, which identify protein-protein interactions, have been used to define the protein interactome for WNV [23,24,25,26] and DENV viral proteins, [23,27,28,29,30,31,32,33,34,35,36,37]. Khadka *et al.* screened all 10 DENV proteins (using multiple bait constructs for each) against a human liver cDNA library to identify 105 proteins as potential interactors [32]. The authors found enrichment among proteins in the complement system (also noted in [37]), the coagulation cascade, the centrosome, and the cytoskeleton (Ibid.). Of 12 of these candidates tested for functional significance by RNA interference-mediated knockdown, six (CALR, DDX3X, ERC1, GOLGA2, TRIP11, and UBE2I) inhibited a DENV replicon (Ibid.). Le Breton *et al.* screened DENV NS3 and NS5 against a cDNA library derived from human liver, spleen, brain and bronchial epithelia, and identified 108 human proteins that interact with NS3 or NS5 or both [34]. Proteins involved in RNA binding, transcription, vesicular transport, and innate immunity were enriched in this group (Ibid.). There was no overlap between the NS3 and NS5 interactomes in the Khadka and Le Breton studies, which suggests caution in interpreting these data. 

Affinity chromatography is another commonly used method to determine protein-protein and RNA-protein interactions *en masse*. Several groups have applied this method to identify host (human and mosquito) proteins that interact with WNV and DENV gene products (both proteins and RNAs) [38,39,40,41,42,43,44,45]. Tandem affinity purification (TAP) was used to discover an interaction between DENV2 NS4A and the human RNA binding protein PTBP1, which was shown to be required for efficient propagation of the virus [38]. Although its precise role in viral replication has not been determined, evidences suggested that PTB was involved in negative strand RNA synthesis [31]. Since WNV and DENV are ribonucleoprotein (RNP) complexes, it follows that many critical interactions take place between host RNA binding proteins (RBPs) and DENV RNAs (genome, antigenome and the sub-genomic flaviviral RNA (sfRNA) [46]. RNA chromatography utilizing biotinylated RNAs, RNAs covalently linked to beads or RNA aptamer affinity matrices have been used for enrichment of viral RNA-protein interactions [41,42,45]. Most of these reports have focused on sequences in the 5' and 3' untranslated regions (UTRs), which control the stability and translation of the viral mRNA, and the formation of the sfRNA [46,47,48,49,50]. One study identified the Y box-binding protein-1 (YB-1) and the heterogeneous nuclear ribonucleoproteins (hnRNPs)—hnRNP A1, hnRNP A2/B1, and hnRNP Q- as 3' UTR interactors and showed that YB-1 has antiviral activity [42]. Using tobramycin aptamer RNA affinity chromatography coupled with quantitative mass spectrometry, Ward *et al.* (2011) identified scores of proteins that interact with 5' and 3' UTR DENV-2 sequences, including the DEDD-box helicase DDX6, the 3'–5' exonuclease ERI3, which was also identified using functional genomics (see below), and stress granule associated proteins G3BP1, G3BP2 and CAPRIN-1 [45]. HnRNP A1, A2 and U were found in both studies, however, hnRNP proteins are very abundant and the relevance of the interactions requires further study [42,45]. 

### 2.3. Functional Genomics

Currently the *en masse* functional analysis in human cells (also true in mosquito cells) involves either gain of function screens accomplished by overexpressing cDNAs, or more commonly, RNAi-mediated loss of function screens (using dsRNA, siRNA or shRNA libraries). There has been one published genome-scale screen for human host factors of WNV [51] and one for dipteran host factors for DENV-2 [52], and several smaller scale screens for DENV human host factors [51,52,53,54]. Krishan *et al.* (2008) provided the first look at the extensive array of host factors that impacted WNV replication and tested this set of gene products for their impact on DENV [51]. Sessions *et al.* (2009) adapted DENV-2 for growth in drosophila cells and screened a dsRNA library for insect host factors in insect cells. The human homologs of the identified candidate factors were tested for an effect on DENV-2 replication revealing remarkable conservation for required factors in the human and mosquito hosts [52]. Several groups have carried out more focused screens using DENV replicons to assay replication, avoiding early viral entry and uncoating or viral assembly and release factors, and targeting subsets of gene products [53,54]. The available data suggest that various screening approaches undertaken so far have not revealed the full spectrum of host factors impacting on flaviviruses, and there remains significant room for future focused and genome-scale screens to contribute meaningfully to the database of WNV and DENV host factors. 

There is important debate regarding the reproducibility and on-target accuracy of RNAi screens. It should be acknowledged that some genes cannot be knocked down efficiently and that some genes may have redundant functions. Careful experimentation can ensure significant reproducibility, as observed in the case of a human genome-scale RNAi screen for host factors required for replication of yellow fever virus (17D vaccine strain) (YFV-17D) [55,56]. For a careful discussion of these considerations and technical details of running and analyzing these screens we refer the reader to Barrows *et al.* [55]. The overall accuracy of screens has been difficult to assess and is likely variable depending on how noisy the screens are. Several parameters should be considered: first, primary screens should interrogate each gene product with more than one dsRNA/siRNA/shRNA (or sets of these). Such an approach would reduce the number of potential false positives resulting from the screening. Second, an RNAi independent methodology should be used to support the findings of the screen (e.g., genome editing with RNA guided endonucleases [57]). 

### 2.4. Genome-Wide Associations Studies

Recently, a genome-wide association study (GWAS) coupled with validation of significantly associated markers was performed to identify genes associated with dengue shock syndrome (DSS), by comparing pediatric patients with controls. This study identified association of major histocompatibility complex (MHC) class I polypeptide-related sequence B (MICB), and phospholipase C, epsilon 1 (PLCE1) with DSS [58], and a subsequent study extended the findings to less severe pediatric dengue fever [59]. Another study identified an association between SNPs in JAK1, which is critical for IFN signaling, and DHF [60]. A comprehensive GWAS study in the context of WNV infection is yet to be reported. An early study using 501 human seroconverts revealed that a single nucleotide polymorphism (SNP) in the OAS1 gene increases the susceptibility of humans to WNV [61]. Similarly, another recent study involving 753 WNV seroconverts identified SNPs in IRF3, MX1 and OAS1 that can affect the host susceptibility to WNV [62]. A third study reported that a mutation in chemokine receptor CCR5 is associated with human resistance to WNV infection [63]. It is conceivable that with the availability of large-scale GWAS studies, our understanding of the role of host factors in the susceptibility and effective host response to WNV infection will expand further.

## 3. A Summary of the Current State of Anti-Flaviviral Therapeutics

As there are no approved therapies to inhibit or prevent flaviviral infections such as those caused by WNV and dengue virus, intensive research using three general approaches have been considered to attain this goal: inhibition of viral gene products (proteins and much less well studied viral RNAs); inhibition of host dependency factors; and modulation of host restriction factors [64,65]. 

### 3.1. Targeting Viral Products

There are many compounds targeting flaviviral NS proteins, however, few exhibit pan-flaviviral activities despite significant conservation in the sequence of NS proteins across flaviviruses [66]. The highly conserved NS5 has guanylyltransferase and methyltransferase (MTase) activities, which cap the 5' end of flaviviral RNAs, and an RNA-dependent RNA polymerase (RdRp) activity, which synthesizes viral RNAs. The Mtase domain is a reported target for 2-thioxothiazolidin-4-ones, S-adenosyl-L-homocysteine (SAH) derivatives, aurintricarboxylic acid, and to a lesser extent Ribavirin triphosphate (RTP) and 1-beta-d-ribofuranosyl-3-ethynyl-[1,2,4]triazole (ETAR). The 2-thioxothiazolidin-4-ones reduce viral replication [67] and aurintricarboxylic acid derivatives block nucleic acid-protein interactions [68], but both classes are relatively non-specific. Other Mtase inhibitors are nucleoside analogues (ribavirin and ETAR) or mimic a known endogenous cellular substrate (e.g., SAH). NS5 RdRp inhibitors include both nucleoside and non-nucleoside compounds. Two well-studied nucleoside inhibitors, NITD008 (and the similar analog, NITD449) and 7-deaza-2'-C-methyladenosine, are adenosine mimetics. Although NITD008 and NITD449 are potent inhibitors of flaviviruses *in vitro*, they caused severe toxicity in animals when administered for two weeks [69,70]. One allosteric, non-nucleoside inhibitor of the DENV RdRP is N-sulfonylanthranilic acid, a compound believed to affect the elongation phase of RNA synthesis and for which no toxicity data is published [71]. The flaviviral NS3 protein has two enzymatic activities, a serine protease (with assistance from NS2B) and an RNA helicase. The NS2B-NS3 serine protease has been shown to be a druggable target [72,73], as first proposed by Padmanabhan and colleagues [74]. Although a class of guanidinylated 2,5-dideoxystreptamine compounds was identified to inhibit DENV protease activity in assays that used purified protease, they did not show any anti-dengue viral activity in cells [66]. The cyclohexenyl chalcone derivatives of natural flavonoids were also found to inhibit dengue NS3 protease [75]. Recently an uncharged tetrapeptide, WYCW-NH2, has been shown to potently inhibit DENV1-4 proteases [76] and functionalized 1,2-benzisothiazol-3(2H)-one-1,3,4-oxadiazole hybrid derivatives were shown to inhibit DENV2 and WNV proteases [77]. Hepatitis C virus (HCV) (not a flavivirus but a member of the *Flaviviridae* family) helicase has been inhibited with quinolone derivatives, which also inhibit the HCV RdRp activity [78] and some of these derivatives could potentially inhibit DENV helicases [79]. Recently, the anti-helminthic drug Ivermectin was shown to inhibit YFV helicase and, to a lesser extent, that of other flaviviruses [80]. 

Whereas most of the aforementioned studies used small molecule inhibitors, other reports describe the use of morpholinos to inhibit DENV2 growth in tissue culture [81] and of RNA interference to block WNV and JEV encephalitis in mice [82]. Therapeutic antibodies have also been proposed in prophylaxis and treatment [83]. While we believe these methodologies hold promise we have decided to focus here on small molecule inhibitors. 

### 3.2. Targeting Host Dependency Factors

As discussed above, flaviviruses require the activity of scores of host factors to propagate efficiently, allowing drugs that inactivate these factors to be used to combat infections. This general approach has gained recent momentum in part because of the successful use of CCR5 antagonists in HIV-1 infected individuals [84]. While several host factors are either proven or implicated in the infection of DENV and WNV (reviewed in [5,6]), only a subset of them have inhibitors already available. Our review will highlight only those host factors against which inhibitory drugs are available, or are previously demonstrated to be readily amenable for drug targeting. Various studies conducted so far to evaluate the suitability of host molecules as therapeutic targets against flaviviral infections are described below, classified based on both viral and host cell perspectives. Some of the host targeting drugs tested against WNV and DENV are listed in Table 1.

**Table 1:** List of selected host targeting drugs tested against WNV and DENV.

Tested host-targeted flavivirus inhibitors	Cellular target/stage in viral lifecycle	Virus	References
**Host metabolic processes**			
Cerulenin	Fatty acid synthases	DENV, WNV	[85,86]
C75	Fatty acid synthases	DENV, WNV	[85,86]
Lovastatin	HMG CoA reductase	DENV, WNV	[54,87,88]
U18666A	Cholesterol transport	DENV	[89]
25-hydroxycholesterol		WNV	[87]
Methyl b-cyclo Dextrin	Cholesterol transport	DENV, WNV	[87]
ETAR, Mycophenolic acid, Ribavirin, ribavirin triphosphate,	IMP dehydrogenase	DENV, WNV	[90,91,92]
Brequinar	Dihydroorotate dehydrogenase	DENV, WNV	[93]
6-Azauridine	OMP decarboxylase	WNV	[91]
kotalanol	α-glucosydase	DENV	[94]
Castanospermine	α-glucosydase	DENV	[95,96]
CM-9-78	α-glucosydase	DENV	[97,98]
CM-10-18	α-glucosydase	DENV	[97,98]
Cyclosporine	Peptidyl-prolyl isomerases	DENV, WNV	[99]
IU1	USP14	DENV	[100]
MG-132, ALLN	Proteasome	DENV, WNV	[13]
AZD0530	Fyn and c-Src kinases	DENV	[101,102]
Dasatinib	Fyn and c-Src	DENV	[101,102]
SFV785	NTRK1/ MAPKAPK5	DENV	[103]
SDM25N, Naltrindole	δ opioid receptor	DENV	[104]
Narasin	Ionophore	DENV	[105]
Ivermectin	Importin α/β	DENV	[80]
**Viral entry**			
Heparin/Highly sulfated heparan sulfate	Entry	DENV	[106,107]
Chondroitin sulfate E	Entry	DENV	[108]
Fucoidan	Entry	DENV	[109]
κ/ι/ν-carrageenan G3d	Entry	DENV	[110]
DL-galactan hybrid C2S-3	Entry	DENV	[110]
Pentosan polysulfate	Entry	DENV	[111]
Surami	Entry	DENV	[111]
PI-88	Entry	DENV	[111]
Neolactotetraosylceramide	Entry	DENV	[106]
**Immunity targeting**			
Gama-interferon	Immune	YFV	[112]
Type I interferons	Immune	DENV, WNV	[113,114,115,116,117]
Poly (I:C)	Immune	WNV	[118]
Autophagy inducing peptide	Autophagy	WNV	[119]
3-Methyl adenine	Autophagy	DENV	[53,120,121]
**Pathogenesis targeting**			
UK-74,505	Antagonist of PAFR	DENV	[122]
PCA-4246	Antagonist of PAF	DENV	[122]
Anti-IL10	Pathogenesis	WNV	[123]
NS-398	COX-2	WNV	[124]
GM6001	Metalloproteinases	WNV	[125]
Cromolyn	Mast cell	DENV	[126]
Ketotifen	Mast cell	DENV	[126]
Montelukast	Leukotriene receptor antagonist	DENV	[126]

#### 3.2.1. Host Metabolic and Post-Translational Modification Pathways as Druggable Anti-Flaviviral Targets

It has become increasingly evident that cellular metabolic pathways are heavily co-opted by flaviviruses to successfully infect host cells [5,87]. This reliance of viruses on host metabolites also expands the arsenal of potential host targets for therapeutics development against flaviviruses. 

Lipid metabolism is tightly associated with flaviviral infections. For example, cholesterol is essential for the infection of cells by both WNV and DENV. It was found that treatment with lovastatin, the widely used cardiovascular drug that inhibits HMG CoA reductase (catalyzes the committed step in cholesterol biosynthesis), blocks both WNV (Kunjin strain) [87] and DENV infection [54,88] *in vitro*. The drug methyl-β-cyclodextran that sequesters cholesterol, and 25-hydroxycholesterol which affects the cholesterol transcription factor functioning, were also found to reduce WNV infection *in vitro* [87,127]. Similarly, methyl-β-cyclodextran and cholesterol transport inhibitor U18666A were also found to interfere with DENV infection [89,128]. Furthermore, earlier studies have demonstrated that the fatty acid synthesis pathway is also an attractive target for interfering with flaviviruses. Treatment with cerulenin and C75, two inhibitors of fatty acid synthases (FASN), drastically reduced the cellular infection of both DENV and WNV, *in vitro* [85,86]. All these studies collectively underscore the important requirements of various lipid metabolic pathways and metabolites in flaviviral infection. However, despite the extensively studied *in vitro* anti-flaviviral effects of various drugs interfering with lipid metabolism, and approved use of statins in humans, no *in vivo* study has yet been reported evaluating their anti-flaviviral therapeutic potential in either animal models and humans.

Another well-known metabolic target against flaviviruses is nucleotide metabolism. Ribavirin, ribavirin triphosphate (RTP) and ETAR are believed to be inhibitors of inosine-5'-monophosphate dehydrogenase (IMPDH) [90,97]. Ribavirin is used clinically for the treatment of HCV, but always in conjunction with pegylated interferon. Ribavirin exhibits anti-flaviviral effects in cell culture models against both WNV and dengue virus, but results in mouse models have always been poor [91,92,129]. ETAR is a close structural analog to RTP and presumably has the same mode of action in DENV models [130]. As noted above, these nucleoside/nucleotide analogues are thought to have direct inhibitory activities on flaviviral enzymes. The drug Mycophenolic acid, a potent inhibitor of both WNV and DENV, is proposed to exert its antiviral action by inhibiting the enzyme inosine monophosphate dehydrogenase [91,92,129]. Similarly, Brequinar, an inhibitor of dihydroorotate dehydrogenase, a key enzyme needed for *de novo* pyrimidine biosynthesis, was shown to inhibit both WNV and DENV, *in vitro* [93]. In addition, 6-azauridine, inhibitor of orotidine monophosphate decarboxylase, involved in pyrimidine biosynthesis, was also found to control WNV infection in cell culture [91]. Thus, several drugs that target key components of nucleotide metabolism offer promise as anti-flaviviral therapeutics. 

In addition to the above-described well-documented role of lipid and nucleotide metabolic pathways in flaviviral infection, several aspects of post-translational modifications (e.g., glycosylation) have also been demonstrated as drug targets against flaviviral infections. Among these, the carbohydrate modification pathways have been extensively investigated in the context of flaviviral infections. An α-glucosidase inhibitor, castanospermine, was found to be effective in blocking dengue virus, though not WNV, both *in vitro* and *in vivo* in mice [95,96]. The 6-*O*-butanoyl derivative of castanospermine (called celgosivir) has been recently tested in human as a therapy against dengue virus infection [131]. Separate studies also reported additional classes of α-glucosidase with antiviral roles against DENV. It was shown that the sulfonium-ion α-glucosidase inhibitors kotalanol and its de-O-sulfonated derivative can also interfere with DENV infection [94]. The α-glucosidase substrate mimics CM-9-78 and CM-10-18 were also found to efficiently inhibit DENV infection *in vitro*, and reduce viremina as well as DENV infection induced disease and death in mice [97,98]. Interestingly, co-administration of a sub-effective dose of CM-10-18 with ribavirin resulted in a major reduction of viremia [97]. Collectively, all these studies underscore the value of host cellular metabolic pathways as targets for interference with flaviviral infection. 

Several additional cellular processes involved in protein processing are also identified as potential host targets against flaviviral infection. One study reported that the inhibition of the cyclophilin family of peptidyl prolyl isomerases using cyclosporine interferes with the replication of both WNV and DENV, at sub-cytotoxic concentrations [99]. Mechanistically, cyclosporine was found to disrupt the interaction between WNV NS5 protein and cyclophilin A, which was needed for successful infection [99]. Other studies have identified the ubiquitin proteasome pathway as druggable host targets against flaviviruses. Nag *et al.* (2012) identified IU1, a small molecule inhibitor of the ubiquitin specific protease USP14, as a potent inhibitor of DENV infection [100]. An earlier study revealed that DENV infection is sensitive to proteasome inhibition using the drugs MG-132 and ALLN [13]. Similarly, the replication of WNV was also sensitive to proteasome inhibition [132]. Because drugs targeting the proteasome are already in human clinical use against other medical conditions, this is an avenue that warrants further study. 

#### 3.2.2. Druggable Host Dependency Factors Identified through Screening Approaches

Several studies have taken a drug-based discovery approach to identify host molecules that can be exploited to control flaviviral infections. Excellent studies aimed at discovering host factors that can be targeted to control flaviviral infection have in fact revealed several such druggable candidates. Yang and colleagues focused on the role of kinases in dengue infection, and identified the kinases Fyn and c-Src (using the drugs AZD0530 and Dasatinib) as druggable host proviral factors [101,102]. Our own previous study revealed that the kinase inhibitor SFV785 targeting NTRK1 and MAPKAPK5 kinases potently inhibited DENV infection [103]. Another recent study that screened the NIH Clinical Collection library of drug-like molecules identified the δ opioid receptor antagonist SDM25N, and the structurally analogous naltrindole, as notable inhibitors of dengue virus [104]. In addition, the ionophore narasin was identified to have appreciable activity against dengue virus [105]. Another recent study found that inhibiting importin α/β-mediated nuclear transport using ivermectin interferes with dengue virus infection [133]. A relatively recent study evaluated the anti-dengue effect of a collection of 5,632 drugs that also included well-characterized bioactives and U.S. Food and Drug Administration-approved drugs [134]. Interestingly, more than half of the 73 drugs that were identified as inhibitors of dengue virus in this screen are known to target a variety of cellular proteins. These results clearly imply that there are several host targets that can be targeted to treat DENV and WNV infection, through re-purposing of drugs previously approved for human use. 

#### 3.2.3. Druggable Host Targets Implicated in the Cellular Entry of Flaviviruses

A major initial step in viral infection is the entry into target host cells. Therefore the cellular entry stage of viral infection has always been an attractive area of antiviral research. The dengue and West Nile viruses are no exception to this trend.

Our understanding of the host cellular receptors and co-receptors of both WNV and dengue virus entry is incomplete, and no definitive receptor has been established to date for either of these viruses. More information is available for the entry requirements of dengue virus than WNV. Several proteins have been reported as putative host targets needed for the cellular entry of dengue virus [135]. These include the mannose receptor [136], HSP90/70 [137], GRP78 [138] and the laminin receptor [139]. A recent study revealed a major role for the TIM and TAM family of receptors in mediating cellular entry of dengue virus [140]. DENV is well characterized to require cell surface carbohydrates for attachment to the cell surface, offering a potential area for therapeutic intervention [141]. Glycosaminoglycans are linear polysaccharides comprised of disaccharide units of an acetamido sugar (*N*-acetyl-D-glucosamine or *N*-acetyl-D-galactosamine) and a uronic acid (D-glucuronic or L-iduronic acid) or D-galactose units. Studies showed that cell surface glycosaminoglycan modifications (e.g., heparansulfate, chondroitin sulfate E) are important requirements for the infection of dengue viruses. Particularly, highly sulfated heparan sulfate expression was shown to be essential for dengue viral entry [106,142]. In agreement with this, many glycosylated cell surface proteins such as lectins (e.g., DC-SIGN, L-SIGN, mannose receptor) are shown to be important for dengue entry [143,144]. A variety of GAGs or mimics have been tested as (competitive) inhibitors of dengue virus entry. One of the earliest studies showed that incubation of cells with heparin and highly sulfated heparan sulfate strongly inhibited cellular infection of dengue virus serotype 2 [142]. Another investigation observed that treatment with chondroitin sulfate E inhibited the early steps of cellular infection of all four serotypes of dengue viruses [108]. Several other charged carbohydrates were also found to interfere with dengue virus infection. It was reported earlier that fucoidan, a sulfated polysaccharide from the marine alga *Cladosiphon okamuranus*, potently interfered with the infection of dengue virus serotype 2, but not other dengue serotypes [109]. The antiviral ability of fucoidan was determined to depend on its sulfation. A related study identified that sulfated polysaccharides κ/ι/ν-carrageenan G3d and the DL-galactan hybrid C2S-3 isolated from the seaweeds (*Gymnogongrus griffithsiae* and *Cryptonemia crenulata*) displayed predominant inhibitory activity against dengue virus, primarily to serotype 2 [110]. Another interesting study compared the anti-dengue viral activity of sulfated polysaccharide mimetics pentosan polysulfate, suramin, and PI-88 *in vitro*, and found that they all were active against dengue virus with the first one being most potent, followed by the other two, in decreasing order [111]. However, in mice, only PI-88 retained antiviral activity significantly. Apart from glycoaminoglycans, the glycosphingolipid neolactotetraosylceramide (nLc4Cer) was also identified as an important host cell surface component interacting with all four serotypes of the virus [106]. Synthetic analogues of nLc4Cer with altered structure strongly inhibited the infection of DEN2 virus, *in vitro* [106]. As summarized here, significant progress has been made to develop inhibitors of dengue virus by targeting the carbohydrates involved in cellular viral entry. It is reasonable to predict that discovery of additional host molecules that mediate the cellular entry of WNV and DENV will open up new targets and approaches to inhibit the early steps of infection.

### 3.3. Targeting Host Restriction Factors

The third approach involves modulation of the host response. Diverse arms of the immune response contributes to the control of flaviviral infections; however, the focus of this review will only be on those host immune factors and mechanisms that offer non-traditional and emerging avenues for intervention. The traditional aspects of immune control such as vaccines are reviewed elsewhere [145,146,147,148,149]. Broadly, we will discuss two aspects of host immunity that hold the potential to emerge as novel antiviral therapeutic targets: modulation of (1) host restriction (antiviral) factors/mechanisms, and (2) host factors that are involved in the immunopathogenesis. 

#### 3.3.1. Targeting Host Restriction Factors to Control WNV and DENV Infection

The best-studied antiviral systems are the pattern recognition receptors and interferons [150]. Type I interferons play critical roles in the immune control of all studied flaviviruses including WNV and DENV [113,114,115,116,117]. Yet, the direct application of interferons to treat infection by these viruses has not been extensively investigated *in vivo*. In an early trial of Type II interferons (IFNs) for prophylaxis and treatment of yellow fever virus (YFV) infection, we showed that IFN-γ treatment could ameliorate the severity of YFV in Saimiri sciureus (squirrel monkeys) and Macaca mulatta (rhesus monkeys), but did not change mortality in the latter [112], a model of severe YFV. Beginning with our earlier investigation to evaluate the efficacy of IFN-γ against YFV infection, a limited number of additional studies have been performed on other flaviviruses. A clinical report revealed that two WNV infected human subjects who were experimentally treated with recombinant interferon-α (IFN-α2b) showed improved outcomes after WNV infection [151]. In another report, however, it was reported that two WNV infected patients did not respond well to IFN-α2b therapy [152]. The therapeutic clinical application of interferon to treat WNV infection has not been tested using large human WNV patient cohorts, and hence any conclusion drawn based on these small sample size based earlier studies will be inaccurate. Equally, the use of interferon as a therapy against human dengue viral infections is yet to be evaluated systematically. Encouragingly, administered IFN-α2a was found to reduce dengue viremia in a rhesus monkey infection model, indicating its therapeutic potential in primates [153]. As an alternative to the direct application of interferon in patients, recently, another approach involving activation of the interferon system by stimulating the PRRs with their ligands (ligand-mimics), has emerged. A study in mouse models of WNV infection using PRR stimulation with interferon inducing genomic RNA fragments of foot and mouth disease virus (5' non-coding region and IRES), and to a lesser extent with double stranded RNA mimic poly(I:C), served to control the viral infection and host death [118]. In a related context, the recent identification of small molecular weight compounds that are potent inducers of interferons offers the promise of a new class of host-targeting antivirals [77]. Although these were tested only against influenza infection, their potential use as antivirals against flaviviral infection will be worth evaluating. The primary advantage of this paradigm is that it offers immense potential to exploit the host’s very potent antiviral mechanisms to control viral infection, and hence will likely have less toxicity. Thus, the evolving picture indicates that interferons and interferon-activated pathways are under-investigated as potential therapeutics against flaviviruses.

Interferons exert their antiviral functions through a diverse array of interferon-stimulated genes (ISG) and mechanisms, which could also be developed as therapeutic strategies [154]. There are at least two general ways by which ISGs can be envisioned to be developed as therapeutic antiviral targets. They can be either selectively modulated to enhance their antiviral function by a stimulant (e.g., small molecules that can activate ISG), or by developing drugs that phenocopy the mechanism of action of ISGs (e.g., small molecules that mimic the antiviral action mechanism of a particular ISG). However, the species specificity associated with their antiviral action has to be taken into account while exploiting the ISGs to control viral infections. Both WNV and DENV are known to be sensitive to the endoribonuclease RNase L, which is activated by 2'–5' oligoadenylate synthesized by 2'–5' oligoadenylate synthetase [155,156]. Although not yet tested, 2'–5' oligoadenylate holds the potential to be developed as a therapeutic to inhibit flaviviruses through RNase L activation. Supporting this notion, indeed, lipidated variants of 2'–5' oligoadenylate with improved half-life and bioavailability were shown to be effective against influenza virus in cell culture [157]. In this context, it is worth highlighting a related study that identified 25-hydroxycholesterol, synthesized by the ISG cholesterol-25-hydroxylase, as a potent inhibitor against several RNA viruses [158]. As mentioned earlier in this review, 25-hydroxycholesterol can inhibit infection of cultured cells by WNV [87]. 

Autophagy is another host process that can be exploited to limit flaviviral infections. Autophagy is a complex cellular process involved in both cellular component degradation and recycling, and is also integrated with a wide variety of physiological processes. Not surprisingly, autophagy is also associated with viral infections, and is known to serve as either a proviral or an antiviral immune mechanism. While autophagy did not impact *in vitro* WNV infection [159], successful infection of human cells by DENV was shown to require the induction of autophagy [53,120,121,160]. Interestingly, it has been shown that an autophagy inducing peptide protects mice from lethal WNV challenge, likely promoting viral killing through autophagy [119]. 

In summary, several excellent avenues exist to target non-traditional aspects of host antiviral immunity to mitigate viral infections. Future attention to exploit their human therapeutic potential and clinical application warrants extensive validation *in vivo* using appropriate animal models.

#### 3.3.2. Targeting Host Factors Mediating Pathogenesis

Diverse sets of immune and non-immune host molecules are involved in disease pathogenesis during viral infections. Targeting these molecules may help to control infection-triggered worsening of physiological homeostasis, in turn enabling the body to control viruses by itself. There are several examples for these, relevant to flaviviruses. Several host targets including cytokines have already been tested to alleviate pathogenesis of flaviviruses. The platelet-activating factor receptor (PAFR) has been documented to play a key role in dengue virus pathogenesis. A recent report describes successful use of two platelet-activating factor receptor antagonists to decrease thrombocytopenia, vascular permeability and mortality in a mouse model of dengue infection [122]. 

Several excellent studies have also revealed multiple host targets to mitigate WNV pathogenesis. In one study, targeting the cytokine macrophage migration inhibitory factor (MIF) with both a small molecule antagonist and an antibody was found to protect against WNV pathogenesis [161]. Similarly, neutralization of IL-10 with antibody protected mice from WNV infection induced lethality [123]. In a related context, a previous study found that inhibition of Cyclooxygenase-2 (Cox2) reduces neuroinflammatory cytokines in brain cells, *in vitro* [124]. This indicates that Cox2 is a potential host target for controlling neuroinflammation caused by neurotropic flaviviruses such as WNV. Collectively, these studies strongly demonstrate that interfering with the cytokine response may help to alleviate the pathogenesis caused by WNV, and enhance pathogen clearance. 

Viral infections can also cause structural damage to host organ systems, leading to pathogenesis. Therefore any drug that can prevent these infection induced “structural damages” of the host will have great clinical value. For example, a major mechanism by which dengue virus harms the human host is by inducing damage to vasculature, leading to vascular leakage. Recently, both mast cell stabilizing drugs and leukotriene receptor antagonist have been found to be effective to control vascular leakage in mice injected with dengue virus [126]. The WNV infection induces blood brain barrier (BBB) integrity loss, and the matrix metalloproteinases (MMP) have been implicated in this process [162]. Interestingly, treatment with the MMP inhibitor GM6001 protected the integrity of cell-cell junctions of WNV infected brain microvascular endothelial cells *in vitro* [125]. This observation offers the possibility that proteins involved in BBB integrity regulation are potential targets that can be exploited to control pathogenesis.

The above-cited studies argue that targeting of both host restriction mechanisms and pathogenesis processes, perhaps simultaneously, may eventually become part of a comprehensive therapeutic regimen to manage flaviviral infection and disease of humans. However, several of these approaches are still in their development phase, and will have to endure extensive maturation before their practical clinical application can be evaluated in humans. 

## 4. Conclusions and Future Directions

The studies described in this review clearly demonstrated that several host molecules hold the potential to serve as therapeutic targets to treat flaviviral infections. These include both metabolic pathways and non-metabolic targets. While some of these host molecules have already been tested in animal models of infection or humans, most await detailed experimentation and validation. The host-targeting antivirals may not result in rapid formation of drug resistant mutations in the viruses.

Future attempts to develop host-targeted therapeutic approaches to successfully contain the threat of flaviviral infections should take into account at least four general areas of research: (i) Systematic identification and validation of host targets implicated in flaviviral infections. The multitude of flaviviral infection-associated host molecules (proviral and restriction factors) identified through various *en masse* approaches (e.g., RNAi screens) need to be mined and adequately validated to identify candidates with the potential for modulation by drugs. There is also a need for newer discovery studies to identify essential host factors impacting flavivirus infections in the most appropriate cell types (e.g., primary cellular targets of viruses during *in vivo* infection). These genetic study-based identifications of the flavivirus host factors should be complemented with more focused screens and validations using drugs that exclusively target known host molecules. Such an approach will provide us with a wider choice of drug targets and drugs against viruses; (ii) *In vivo* validations. The druggable host target genes that are already identified (as well as the to-be-discovered targets) to regulate viral infection need to be validated using appropriate gene knockout *in vivo* models of infections. Given the current availability of better animal models for WNV (e.g., mouse), one would anticipate faster progress in the case of WNV than for DENV. Similarly, the drugs targeting the identified host factors should also be evaluated using the most suitable animal model that mimics infection induced disease in humans; (iii) Cross-protection. Because different flaviviruses have a significant degree of conservation in their genome, some of the mechanisms involved in their interaction with host cells are also likely to be conserved. Therefore, any drug or drug target identified as useful against a particular flavivirus should be tested against a wide range of medically relevant flaviviruses of the genus *Flavivirus*. This will help to identify pan-flaviviral therapeutics; and (iv) Drugs boosting host innate defenses. Particular emphasis should be given to discover drugs that can augment various innate antiviral mechanisms of cells. In addition to enhancing the generic production of interferon, such drugs should ideally activate specific effector mechanisms of cells against viruses. For example, a drug that can enhance the production or antiviral activity of interferon stimulated antiviral genes may offer better protection from infection, and less toxicity to the host. A sub-category of immune response boosting drugs should also have the ability to attenuate viral interference with host immune defenses. 

In summary, a multipronged approach integrating both host target and host-directed drug discovery will ultimately facilitate the identification of effective therapeutics against flaviviruses. 
